# Research on the Magnetic Properties of High-Saturation Magnetically Induced Alloy Motors under Magnetocaloric Coupling

**DOI:** 10.3390/ma17061274

**Published:** 2024-03-09

**Authors:** Jun Li, Zhiye Li, Yulin Li, Jiahao Ge, Yuxiao Li, Lubin Zeng, Ruilin Pei

**Affiliations:** 1 Department of Electrical Engineering, Shenyang University of Technology, Shenyang 110870, China; lijun@smail.sut.edu.cn (J.L.);; 2 Suzhou Inn-Mag New Energy Ltd., Suzhou 215000, China

**Keywords:** 1J22, motor, magnetic properties, temperature field–electromagnetic field coupling

## Abstract

In the face of the rapid development of the motor industry, some motors with traditional soft magnetic materials can no longer meet the needs of the market. Using new high-saturation magnetic density materials has become a new breakthrough to improve the torque density of motors. Fe-Co alloys (1J22) have high-saturation magnetic induction strength, which can effectively improve the motor’s magnetic field strength and increase its torque density. At the same time, the temperature rise of the motor is also an important factor to consider in the motor design process. In particular, the change in core temperature caused by loss makes the coupling of the internal temperature field and the electromagnetic field of the motor more common. Therefore, it is necessary to test the temperature and magnetic properties of 1J22 together. In this paper, a coupling measurement device for magnetic properties of soft magnetic materials is built, and a 1J22 temperature field–electromagnetic field coupling experiment is completed. It is found that the maximum loss of 1J22 decreases by 4.44% with the increase in temperature; the maximum loss is 6.41% less than that of traditional silicon steel. Finally, a finite element simulation model is built to simulate the actual working conditions of the motor, and it is verified that the magnetic properties of the material at high temperature will have a certain impact on the performance of the motor.

## 1. Introduction

At present, the core material of the stator of the motor is still dominated by silicon steel sheets. With the continuous improvement in people’s requirements for motor performance [1], the motor performance of traditional silicon steel sheet has encountered a bottleneck, and some electromagnetic materials with better performance have begun to be studied and gradually applied to motors. Some scholars have made ultra-high-speed motors by using the characteristics of amorphous high frequency and low loss [2,3], which has higher efficiency than motors made of traditional materials [4]. High-strength steel is also a new material for making high-speed motors [5,6], which resists the centrifugal force caused by the high-speed rotation of the rotor through its ultra-high yield strength [7]. Fe-Co (content of Co is 49~50%) alloys have been used in high-performance motors in recent years. It has a high magnetic induction strength compared to traditional silicon steel materials [8,9]. Therefore, the use of Fe-Co soft magnetic alloy materials instead of traditional materials to produce motors with high power density and high torque density is a practical breakthrough direction. Some scholars have fabricated Fe-Co alloy motors and compared and analyzed the performance of Fe-Co alloy motors and silicon steel sheet motors [10]. The Fe-Co alloy motor has a higher output torque at the same current [11].

Although Fe-Co alloys have the superior electromagnetic properties described above, the magnetic properties before and after annealing are quite different [12]. Different annealing processes also have a great influence on the magnetic properties of the material. By adding the trace element niobium for heat treatment, the processing performance of Fe-Co alloy materials can be improved without affecting their magnetic permeability [13]. Different heat treatment temperatures will also affect the magnetic properties of Fe-Co alloys [14]. Normally below 730 °C, the Fe-Co alloy changes from a disordered bcc structure to an ordered CsCl-type structure [15]. Selecting the optimal heat treatment temperature will increase the saturation magnetic density of the Fe-Co alloy. Moreover, the residual stress of Fe-Co alloys can be effectively reduced using secondary annealing [16]. In general, choosing the appropriate annealing method will further improve the magnetic properties of Fe-Co alloys.

In addition, the core material is an important part of the motor design, and the calculation of the core loss needs to be more precise. Traditional motor design methods pay more attention to the material data at room temperature and when stress-free, but the material itself is susceptible to external factors. Therefore, considering the influence of more external factors offers a huge improvement in the design accuracy of the motor [17]. In the process of continuous operation of the motor, the core material is susceptible to the combined effects of temperature, stress and electromagnetic fields [18]. Compressive stress often causes the deterioration and loss of core materials [19,20,21]. However, temperature is more friendly to core loss. The loss of the traditional silicon steel sheet is affected by temperature, and decreases with the rise of temperature [22,23]. Some scholars have also modified the iron loss model of materials by considering the influence of multiple field factors such as temperature and stress [24,25]. However, these data are often not the same, depending on the material. The loss of high-silicon steel increases with the increase in temperature [26]. The loss of the Fe-Co alloy in the face of compressive stress shows a decreasing trend [27]. However, the variation law of the magnetic properties of 1J22 under the temperature field is not clear.

This article is based on the above research, where the variable temperature test of 1J22 is carried out by using a magnetocaloric coupling device and applied to the motor core. This paper is divided into five parts, the first part of which summarizes the current research on core materials. The second part selects the appropriate heat treatment method and uses magnetic measurement equipment to test the magnetic properties of 1J22. The third part proposes a magnetocaloric coupling device to complete the variable temperature experiment between 1J22 and traditional silicon steel. The change law of magnetic properties of 1J22 under the temperature field was found and the reasons were analyzed. In the fourth part, finite element simulation is used to verify that the loss at different temperatures will have an impact on the design of the motor. Finally, conclusions are drawn in Section 5.

## 2. Material Analysis and Testing

The difference in chemical composition and content in Fe-Co alloys has a great influence on the magnetic and mechanical properties of the material. When the Co content is 35%, the saturation magnetic induction intensity reaches the maximum value of 2.4 T [28]. When the Co content is 50%, the saturation magnetic induction intensity drops to 2.35 T [29], but it is still far more than the traditional silicon steel sheet (1.8 T). The torque of the motor is proportional to the saturation magnetic induction strength of the core material. Therefore, by changing the material of the motor core, the torque density of the motor can be greatly improved. When the Co content is between 26% and 71%, there is an ordered phase structure [30], which leads to an increase in the brittleness of the alloy and increases the difficulty of processing. Generally speaking, 1.5~4% V element is added for annealing to eliminate the brittleness of the alloy, and after high-temperature vacuum annealing, the grains will grow and increase [14].

The working principle of the permanent magnet synchronous motor (PMSM) is through the interaction of the armature magnetic field on the stator with the permanent magnet magnetic field generated by the permanent magnet on the rotor, which generates an electromagnetic force that causes the rotor to rotate. The stator, or rotor, uses soft magnetic materials, which are the magnetic circuits of the magnetic field and determine the magnitude of the magnetic field. Therefore, the magnetic properties of the core material are an important factor affecting the design of the motor. To fully understand the magnetic properties of 1J22 and reduce the error caused by material properties, in this paper, the magnetic properties of 1J22 and traditional silicon steel are tested firstly; 1J22 with 50% Co content was selected as the experimental sample, and the thickness was 0.35 mm. The traditional silicon steel material of the same thickness was selected as the comparison item. The specific material composition is shown in Table 1.

**Figure 1:**
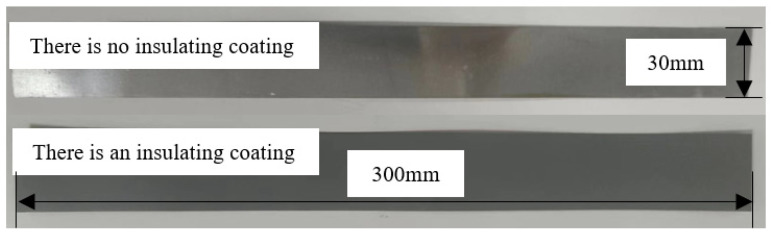
1J22 specimens.

**Figure 2:**
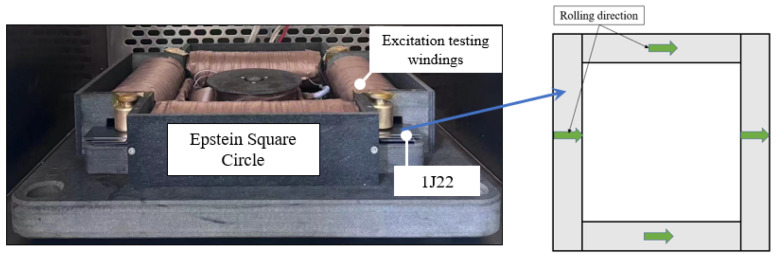
Experimental equipment and methods.

Considering the particularity of the Fe-Co alloy material, 1J22 was first cut and made into a suitable shape. After that, 1J22 was annealed with 1.8% V element at 850 °C. On the one hand, this can eliminate the deterioration of 1J22 loss by cutting, and on the other hand, it can improve the magnetization performance of the material [8]. The 1J22 material was cut into several specimens, half of which were in the direction of the 1J22 rolling, and the other half were in the direction of the vertical rolling. The length was 300 mm, the width was 30 mm, and the total mass was 404.7 g. Since 1J22 has no insulating coating after annealing, 1J22 sprayed insulating solution was selected for insulation. The effect is shown in Figure 1. The experimental setup and methods are shown in Figure 2.

Errors were inevitable during the experiment. To ensure the continuity and authenticity of the experiment, some factors that have little influence on the experiment were ignored, and all experiments were repeated three times to take the average value. The possible errors in the experiment are as follows:

1. In this experiment, the insulating coating was sprayed manually, resulting in an uneven thickness of the 1J22 surface coating.
2. The 1J22 sample was sheared first and then annealed, so the effect of shear stress on 1J22 was minimal. However, traditional silicon steel is annealed first and then sheared, so the experimental results of traditional silicon steel were influenced by shear stress.
3. There are many annealing methods for 1J22, and this paper only selects one of them. Therefore, the experimental results may be different from the results of other literature.

**Figure 3:**
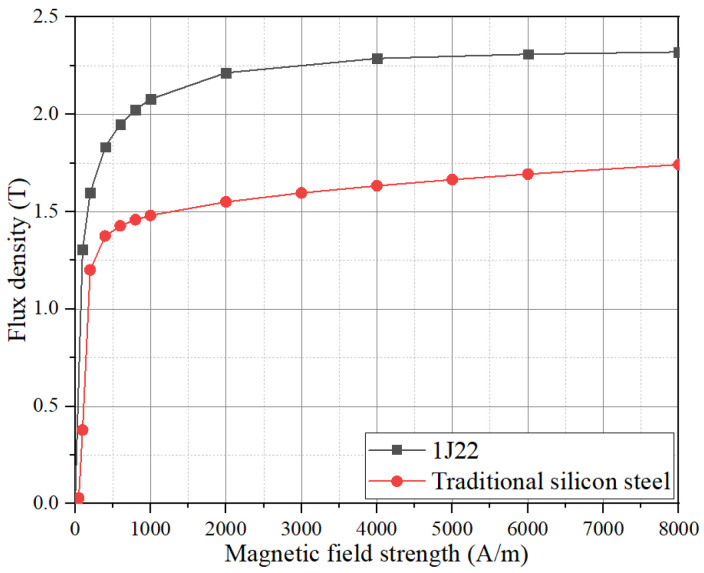
B–H curve of 1J22 and traditional silicon steel.

The B–H curve of 1J22 is shown in Figure 3. The Bs value of J22 after the above annealing treatment is much higher than that of traditional silicon steel, with a peak value of 2.3 T. The peak Bs of traditional silicon steel is only 1.6~1.8 T. This means that the torque can be greatly increased by using 1J22 to make a motor, without considering other magnetic properties. And making a motor with the same performance can greatly reduce the electromagnetic weight of the motor, thereby improving the power density or torque density of the motor.

**Figure 4:**
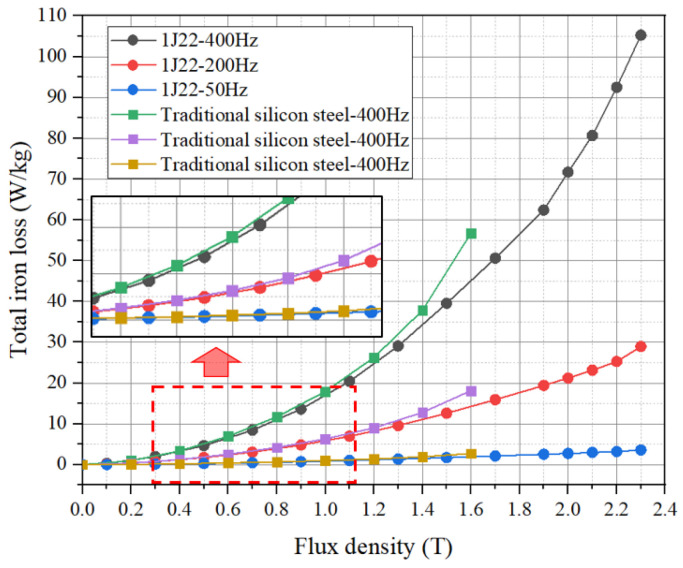
B–P curve of 1J22 and traditional silicon steel at different frequencies.

Since the magnetostriction of 1J22 is extremely sensitive to high frequencies, it is difficult for Epstein square circles to test above 400 Hz. The B–P curve of 1J22 is shown in Figure 4. The loss of 1J22 in the case of medium and low frequency is basically the same as that of traditional silicon steel.

However, the saturation magnetic induction intensity of 1J22 is much greater than that of traditional silicon steel. The peak magnetic induction intensity of the two materials varies greatly. Therefore, when using 1J22 to design motors, attention should be paid to the high loss caused by high magnetic induction intensity.

In addition, the resistivity of 1J22 (0.7~0.75 Ωm) is smaller than that of traditional silicon steel (1.2~1.6 Ωm), and with the increase in frequency, the eddy current loss of 1J22 is greater than that of the hysteresis loss, which accounts for more than the total iron loss. This means that in high-frequency cases, the loss of 1J22 will gradually increase. Therefore, in the process of motor design, the frequency of the motor should be kept in the middle and low frequencies to ensure that the motor has a higher efficiency.

## 3. Magnetocaloric Coupling Experiments

When the machine is operating, the core is in the alternating magnetic field, which will produce hysteresis and eddy current loss. The current flows through the armature windings to produce copper losses, and the surface of the permanent magnet also has eddy current losses due to the action of the magnetic field. These losses are also responsible for the increase in the temperature of the motor.

**Figure 5:**
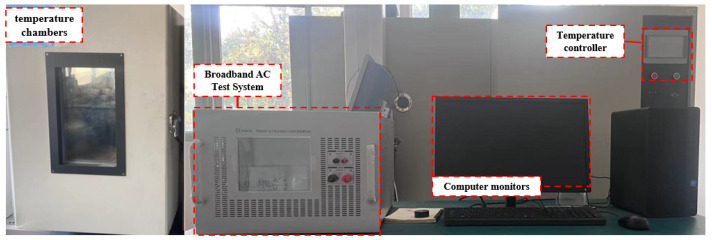
Experimental equipment.

To simulate the above-mentioned working conditions, a coupled test system of the temperature field and the electromagnetic field of the core material was built. Including the electromagnetic field module and the temperature field module, the coupling test system is shown in Figure 5. The coupling experiment of 1J22 and traditional silicon steel at different temperatures was carried out by the temperature module and the electromagnetic module to test the iron loss, and then finish drawing the image. The temperature test points were 0 °C, 25 °C, 50 °C, 100 °C, and the frequency test points were 50 Hz, 200 Hz, 400 Hz.

### 3.1. Results and Analysis

**Figure 6:**
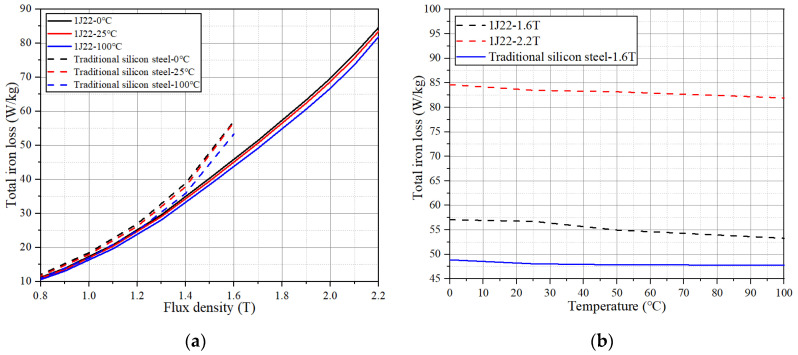
Effect of temperature on the iron loss of two materials. (**a**) Iron loss curves at different temperatures. (**b**) Iron loss varies with temperature.

Figure 6a shows the variation and trend of loss of 1J22 and conventional silicon steel at different temperatures at 400 Hz. Figure 6b shows the change rate of 1J22 and traditional silicon steel at 1.6 T in the face of different temperature losses; the change trend of the two is basically the same, where it increases first and then decreases with the rise of temperature. The loss of traditional silicon steel is more affected by the temperature. The total change of loss from 0 to 100 °C reached the maximum at 1.1 T, decreasing by 6.41%. The loss change trend of 1J22 is basically the same as that of traditional silicon steel, and the maximum point of loss change rate is 1.9 T, which decreases by 4.44%. Then, the loss change rate gradually decreases, and the loss change rate of 2.2 T is 2.23%.

In conventional silicon steel sheets, the conductivity of silicon steel sheets decreases as the temperature rises, whereas conductivity is inversely proportional to resistivity, as shown in Equations (1)–(3). Therefore, when the conductivity decreases, the resistance will increase accordingly, which will reduce the eddy current passing through the silicon steel sheet, resulting in a decrease in eddy current loss [31].

(1) $$\sigma =\frac{1}{\rho }$$

(2) $$R=\frac{\rho L}{S}$$

(3) $$P=\frac{E^{2}}{R}=\frac{E^{2}S\sigma }{L}$$

where P is the eddy current loss, E is the induced electromotive force, S is the cross-sectional area, L is the length, and σ is the conductivity.

As mentioned above, 1J22 has many similarities with traditional silicon steel in terms of loss trends. This is shown in Table 1. Compared with 1J22, traditional silicon steel has the characteristic of having a large amount of Fe elements. Therefore, the temperature affects the conductivity of the Fe element most in traditional silicon steel. Similarly, 1J22 also contains a large amount of Fe element, but 1J22 also has 50% Co element. The Co element has a low thermal conductivity to temperature, which reduces the sensitivity of 1J22 to temperature to a certain extent. Therefore, 1J22 retains the same characteristics as traditional silicon steel in terms of temperature, but the sensitivity is lower than that of traditional silicon steel.

At the same time, as the temperature increases, the coercivity of 1J22 decreases, resulting in the decrease in hysteresis loss of 1J22 [14].

### 3.2. Iron Loss Model Considering the Effects of Temperature

The classic Bertotti iron loss separation model has been widely used to calculate the iron loss of silicon steel sheets. It is mainly composed of hysteresis loss density $P_{h}$, eddy current loss density $P_{c}$ and additional loss density $P_{e}$. Temperature affects hysteresis and eddy current losses in the total losses. In this paper, only the influence of temperature on the iron loss of 1J22 is studied, and the influence of temperature on the additional loss is very small. In subsequent studies, the effect of additional losses was ignored. Under sinusoidal magnetization, the total iron loss density in the silicon steel sheet sample can be calculated by $P_{\mathrm{total}}$ following Equation (5) [32].

(4) $$P_{\mathrm{total}}={\;P}_{h}+P_{e}$$

(5) $$P_{\mathrm{total}}=k_{h}fB_{m}^{\alpha }+k_{c}f^{2}B_{m}^{2}$$

where f is the frequency and B_m_ is the peak value of the alternating magnetic flux density. $k_{h}$ and $k_{c}$ are hysteresis loss coefficients and eddy current loss coefficients, respectively.

From Equation (5), the magnitude of core loss is related to the loss coefficient, magnetic flux density and frequency. Considering this, the effect of different temperatures on iron loss is not the same, but the trend is similar to that of traditional silicon steel. Therefore, the calculation method of the 1J22 loss formula in this paper is similar to that of traditional silicon steel. According to the experimental results in Figure 6, the loss coefficient of 1J22 is obtained by the fitting by least squares method. The iron loss coefficient (400 Hz) of 1J22 at different temperatures is shown in Table 2. In the range of 0~100 °C, the coefficient of hysteresis loss and the coefficient of eddy current loss are changing with the change of temperature.

**Figure 7:**
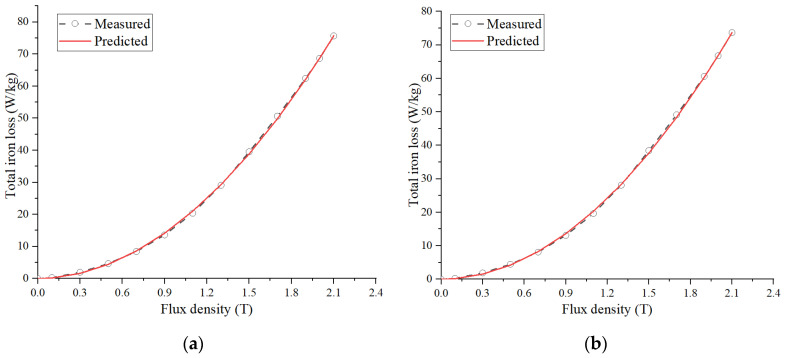
Iron loss curves of various temperatures. (**a**) 25 °C. (**b**) 100 °C.

To verify the accuracy of the model, this paper compares the calculated and measured values of the iron loss model. The results are shown in Figure 7. Due to the close proximity of the loss curves between different temperatures, two temperatures of 25 °C and 100 °C are selected for comparison. The mildness of the two curves is high, and the error between the calculated value and the measured value is less than 2.7%. The error may be in the accuracy of the material test and whether it is within a reasonable range. Therefore, the accuracy of the model is guaranteed.

## 4. Finite Element Simulation of Electric motors

### 4.1. Electromagnetic Field Analysis

**Figure 8:**
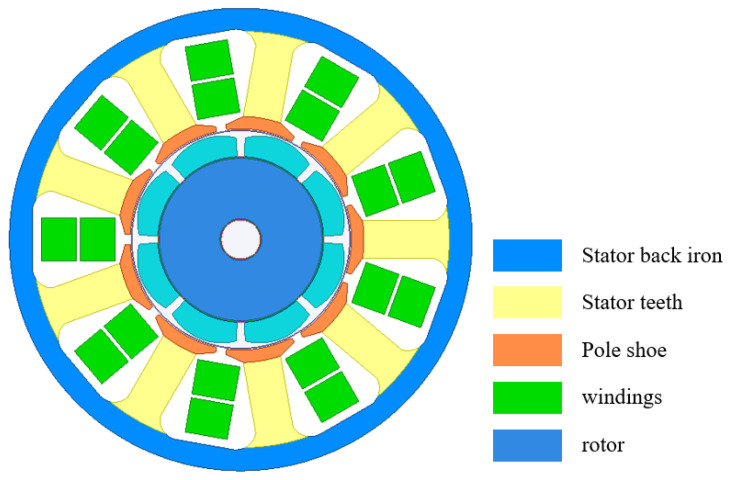
PMSM simulation model.

Based on the above analysis, the motor designed in this paper is a permanent magnet synchronous motor, and the structure is shown in Figure 8. The PMSM adopts an 8-pole and 9-slot structure, and the rotor topology is surface-mounted. The performance parameters of the motor are shown in Table 3.

**Figure 9:**
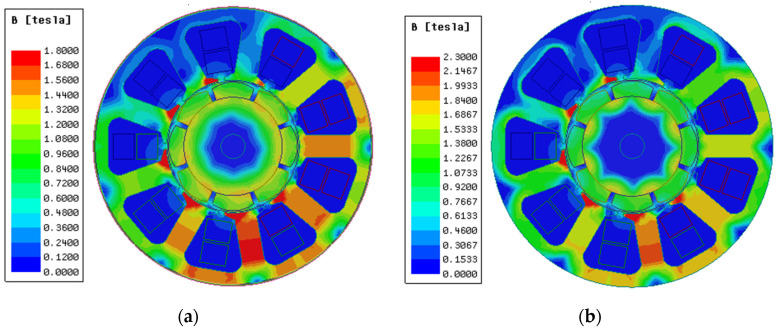
Motor magnetic density cloud diagram. (**a**) Saturated magnetic density of traditional silicon steel. (**b**) 1J22 unsaturated magnetic density (UMSD).

Figure 9 shows the magnetic dense cloud diagram of the two materials. Due to the narrowness of the teeth, it is the main magnetic circuit in which the armature magnetic field interacts with the rotor magnetic field. Therefore, the PMSM saturation magnetic density point first appears in the teeth of the model, while the yoke of the motor is more friendly than the tooth, due to the wide magnetic circuit and no interaction with the rotor magnetic field. Figure 9a shows under the working condition of 6000 rpm and current of 14 A, the use of the traditional silicon steel motor model is more likely to cause magnetic saturation. The saturation degree of the motor tooth and yoke is between 1.5 and 1.8 T, which has reached the limit of material testing. It has become very difficult to increase the torque of the motor.

**Figure 10:**
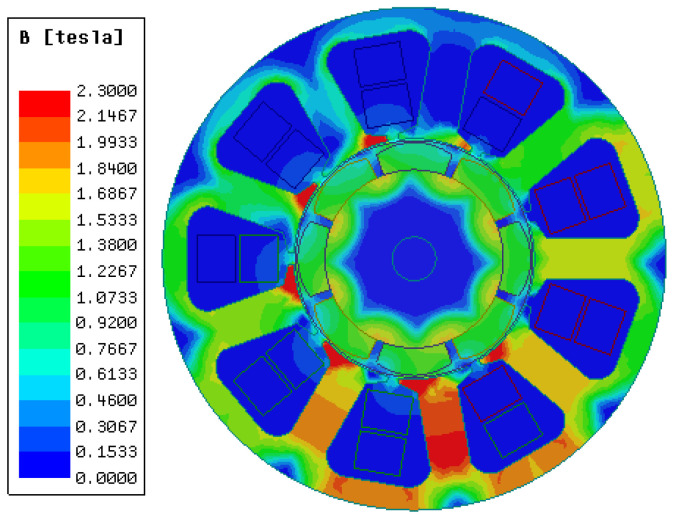
1J22 saturation magnetic dense cloud (SMD).

As shown in Figure 9b, the use of 1J22 can effectively solve this problem. Under the same motor design parameters, using 1J22 as the material of the motor core, the magnetic flux density saturation point of the finite element simulation model is only distributed at the position of the stator tooth pole shoe. The saturation magnetic density of the stator tooth and yoke is only 1.68~2.15 T, which is lower than the saturation magnetic induction intensity of 1J22 by 2.3 T. This shows that there is still room for improvement in torque. By increasing the excitation current, the magnetic flux density of the model can be improved, as shown in Figure 10.

As shown in Figure 10, by increasing the armature current to 16 A, the armature magnetic field inside the motor is greatly improved, and the magnetic dense cloud diagram of the 1J22 motor is greatly improved. The saturation point of the magnetic density of the motor has been transferred from the initial pole shoe position to the teeth of the stator, and the degree of magnetic density saturation of the stator yoke has also changed. The saturation magnetic density of the stator teeth and yoke has reached 1.84~2.3 T, which has reached the peak magnetic density of 1J22. The saturation of the magnetic field of the stator and rotor means that the interactive magnetic field of the motor is also strengthened, and the torque of the motor will also be improved.

**Figure 11:**
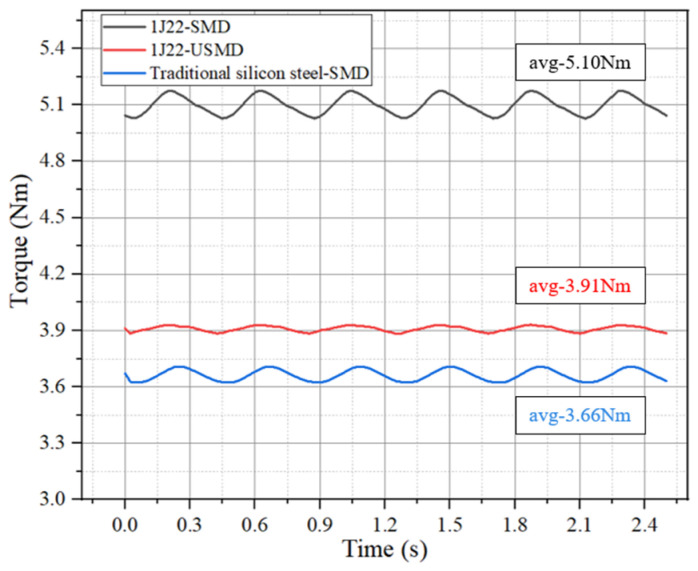
Torque comparison chart.

As shown in Figure 11, when 1J22 is used as the stator core material of the motor, the torque of the motor is significantly improved. When the working current is 12 A, the motor torque of 1J22 is about 6.94% higher than that of traditional silicon steel. When the motor is in 1J22-SMD, the motor torque is increased by 39.34% compared with the traditional silicon steel. This shows that the torque density of the motor can be effectively improved, by giving full play to the characteristics of 1J22 high-saturation magnetic induction intensity without changing other parameters of the motor.

**Figure 12:**
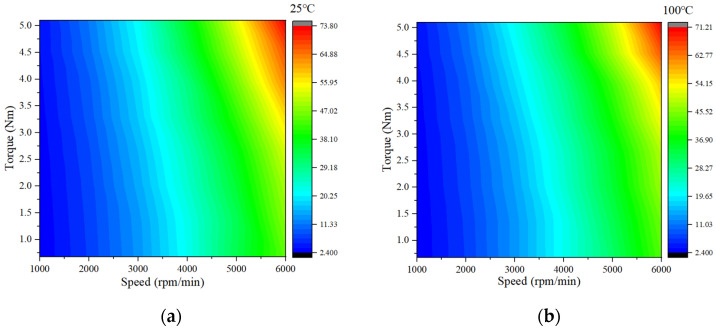
Core loss map. (**a**) 25 °C. (**b**) 100 °C.

At peak speed, the variation in losses under different torques is shown in Figure 12. With the continuous increase in armature current, the loss also increases in the process of increasing torque. In the process of torque change, the core loss of the 1J22 motor is significantly higher than that of the motor at 100 °C under normal temperature conditions. At the highest point, the loss decreased by 6.6%.

**Figure 13:**
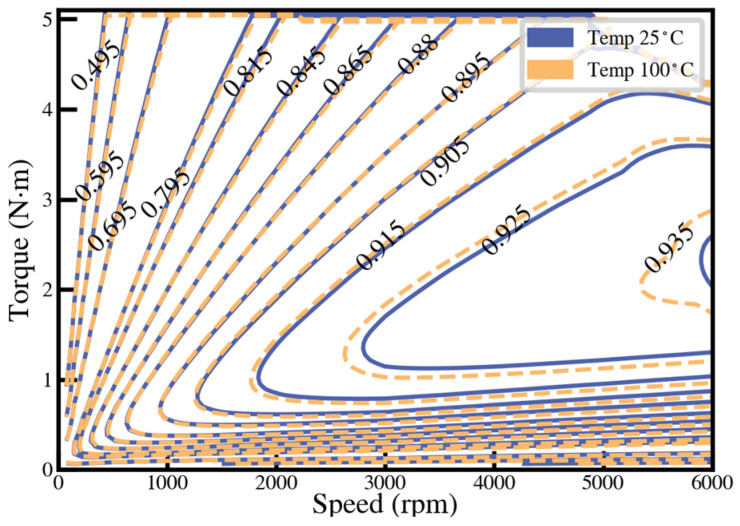
Motor efficiency map comparison of 1J22 at 25 °C and 100 °C.

For PMSM designs, iron loss is an important factor affecting the efficiency of the motor. When the motor is running at peak conditions, the stator and rotor cores are at a high temperature due to losses. In this paper, assuming that the temperature of the stator and rotor core is 100 °C, the motor efficiency MAP plot is plotted at the two temperatures. This is shown in Figure 13, although the maximum efficiency point has not improved much. However, the proportion of the high-efficiency zone of the motor at high temperature is significantly higher than that at normal temperature, and the proportion of the high-efficiency zone greater than 90% is increased by 1.22%. This is because as the speed increases, the torque of the motor decreases, resulting in a larger proportion of core loss. Table 4 shows the detailed parameters.

### 4.2. Temperature Field Analysis

**Figure 14:**
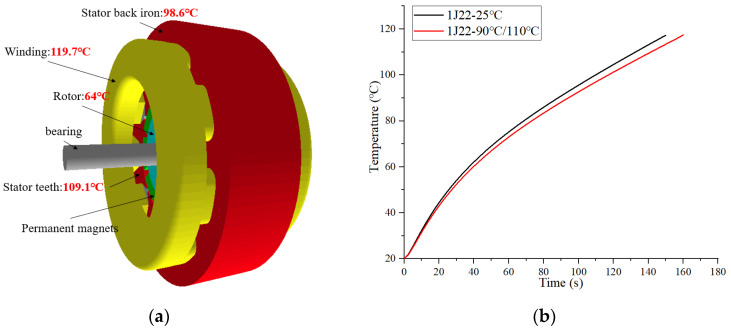
Thermal simulation results (**a**) 3D model (**b**) The curve of motor temperature change.

The principle of temperature rise in PMSM is due to the loss generated by the motor during operation, which is converted into heat, which increases the temperature of the motor. To obtain the temperature rise of the motor more accurately, a finite element 3D simulation model was selected to simulate the temperature field. The cooling method of the motor was set to natural cooling, the ambient temperature was set to 20 °C, and the stator generated heat exchange with the ambient temperature through the casing. The working point of the motor was 6000 rpm/min, the working time was 150 s and the working current was 16 A. The core material was 1J22 at room temperature. The temperature simulation results are shown in Figure 14a. The highest temperature rise point of the core of the motor is at the teeth of the stator, and the maximum temperature is 119.7 °C. This is followed by the stator back iron part, with a temperature of 98.6 °C.

In this paper, only the influence of the change of core material data on the temperature rise of the motor is considered, so the change of copper loss is not considered. According to the simulation results of Figure 14a, the iron loss of 1J22 at 100 °C and 110 °C was re-measured. The remeasured data were brought into each part of the FEM and the finite element simulation calculation was performed again. The results are shown in Figure 14b. Considering the influence of temperature on the core material, the temperature rise of the motor is significantly improved, and the overload time of the motor is extended by 6%.

## 5. Conclusions

In this paper, the iron loss performance of 1J22 under variable temperature is studied. The generation law was analyzed, and an improved iron loss model for 1J22 considering temperature factors was proposed. There is great value in improving the accuracy of the 1J22 motor design, and the specific conclusions are as follows:

1. The iron loss trend of 1J22 material is consistent with that of traditional silicon steel and decreases with the increase in temperature. However, the loss of 1J22 is less sensitive to temperature. The loss of traditional silicon steel decreased the most at 1.1 T, with a decrease of 6.41%. The maximum loss change of 1J22 was 1.9 T, which decreased by 4.44%, and then the loss change rate gradually decreased; the loss change rate of 2.2 T was 2.23%.
2. In the motor design process, the magnetic properties of the core material play a crucial role in the performance of the motor. Through experiments, it is found that the core loss of the 1J22 motor considering the influence of temperature can be reduced by 6.6% at most. With the increase in speed, the proportion of the high efficiency of the motor gradually increases.
3. During the simulation of the motor temperature field, it was found that the motor temperature rise was improved using 110 °C and 120 °C material data. The temperature of the motor rises to 119.7 °C 6% longer than before. Therefore, when designing high-performance motors, it is necessary to consider the change trend of magnetic properties of core materials under the action of temperature, which can improve the calculation accuracy of the model, and increase the reliability of motor design.

## Figures and Tables

**Table 1:** Material content of 1J22 and traditional silicon steel.

Title	C	Mn	Si	Co	V	Other Trace Elements	Fe
1J22	≤0.04%	≤0.3%	≤0.3%	49~50%	1~1.8%	≤0.74%	Allowance
Traditional silicon steel	≤0.02%	≤0.3%	3.2%	/	/	≤0.08%	Allowance

**Table 2:** Loss coefficients.

Temperature	Kh	Kc
0 °C	0.004503564	0.00009990065
25 °C	0.004476332	0.00009769490
50 °C	0.004424972	0.00009716157
100 °C	0.004424972	0.00009716157

**Table 3:** Main parameters of PMSM.

Title	Value	Title	Value
Outer diameter of stator	92	Inner diameter of the stator	44 mm
Poles/slots	8/9	Length	24 mm
Peak rotational speed	6000 rpm/min	Peak torque	3.6 Nm
Peak power	2 kW	Peak current	12 A
Cooling method	Natural cooling		

**Table 4:** Percentage of motor efficiency.

Scheme	1J22-25 °C	1J22-100 °C
Efficiency > 85%	71.86	72.22
Efficiency > 90%	47.50	48.72
Highest efficiency	93.56	93.75

## Data Availability

The data are not publicly available for privacy reasons. The data presented in this study are available from the corresponding author.
